# *Bacillus megaterium* SF185 spores exert protective effects against oxidative stress *in vivo* and *in vitro*

**DOI:** 10.1038/s41598-019-48531-4

**Published:** 2019-08-19

**Authors:** Arianna Mazzoli, Giuliana Donadio, Mariamichela Lanzilli, Anella Saggese, Andrea Maria Guarino, Miriam Rivetti, Raffaella Crescenzo, Ezio Ricca, Ida Ferrandino, Susanna Iossa, Alessandra Pollice, Rachele Isticato

**Affiliations:** Department of Biology, Federico II University, Naples, Italy

**Keywords:** Applied microbiology, Inflammatory bowel disease, Nutritional supplements

## Abstract

Endogenous reactive oxygen species (ROS) are by-products of the aerobic metabolism of cells and have an important signalling role as secondary messengers in various physiological processes, including cell growth and development. However, the excessive production of ROS, as well as the exposure to exogenous ROS, can cause protein oxidation, lipid peroxidation and DNA damages leading to cell injuries. ROS accumulation has been associated to the development of health disorders such as neurodegenerative and cardiovascular diseases, inflammatory bowel disease and cancer. We report that spores of strain SF185, a human isolate of *Bacillus megaterium*, have antioxidant activity on Caco-2 cells exposed to hydrogen peroxide and on a murine model of dextran sodium sulfate-induced oxidative stress. In both model systems spores exert a protective state due to their scavenging action: on cells, spores reduce the amount of intracellular ROS, while *in vivo* the pre-treatment with spores protects mice from the chemically-induced damages. Overall, our results suggest that treatment with SF185 spores prevents or reduces the damages caused by oxidative stress. The human origin of SF185, its strong antioxidant activity, and its protective effects led to propose the spore of this strain as a new probiotic for gut health.

## Introduction

Reactive Oxygen Species, including hydrogen peroxide, superoxide and hydroxyl radicals, are common by-products of the aerobic metabolism of all cells^1^. While at low intracellular concentrations ROS are critical signalling molecules with a role in cell proliferation and survival^2^, their accumulation severely damages DNA, proteins and lipids leading to a variety of chronic diseases, including atherosclerosis, arthritis, diabetes, neurodegenerative, cardiovascular problems and inflammatory bowel diseases^3–7^. All aerobic organisms avoid ROS accumulation with a variety of enzymes (catalase, superoxide dismutase, glutathione peroxidase, and glutathione reductase) and with non-enzymatic molecules with antioxidant activity (glutathione, thioredoxin, Vitamin C, Vitamin E). The production of an excess of ROS induces oxidative stress that plays a crucial role in the establishment of inflammation, thus contributing to the pathophysiology of several diseases^8^. Intestinal epithelial cells are continuously exposed to ROS derived by their own metabolism, by the metabolism of microbes living in the gut and by foods sources^9^. Antioxidant additives contribute to avoid the accumulation of ROS and thus have a protective role against oxidative damages^10^. During the years many natural antioxidant additives have been proposed and tested. Examples include polyphenols (green tea), ginsenosides and, more recently, carotenoids^11,12^.

Intestinal bacteria have a clear role in protecting the animal body against oxidative damages. A study performed with a rat model of diet-induced oxidative stress has shown that an antibiotic treatment, as well as the replacement of the gut microbiota, was able to prevent the oxidative damages in liver and skeletal muscle^13,14^. Therefore, it is not surprising that beneficial bacteria (probiotics) with strong antioxidant properties have been proposed to prevent oxidation of cellular substrates^10^. Examples are species of the *Bifidobacterium* and *Lactobacillus* genera that were shown able to scavenge hydroxyl radicals and superoxide anions *in vitro* and to enhance the action of antioxidant enzymes *in vivo*^15–17^; or cells of a carotenoid-producing strain of *Bacillus indicus*, that was shown to reduce oxidative markers in plasma and liver *in vivo*^18^.

In addition to vegetative cells, also bacterial spores have been shown to have antioxidant properties. Spores are quiescent cell forms produced by over 1,000 bacterial species, mainly belonging to the *Clostridium* or *Bacillus* genera^19^. These microorganisms respond to adverse environmental conditions by producing a highly resistant spore, that can indefinitely survive in the absence of water and nutrient^20^. The dormant spore can, however, germinate originating a vegetative cell able to grow when appropriate conditions are encountered^20^. Spores of several *Bacillus* species are commonly used as probiotics and are known to have beneficial properties^21,22^. An *in vitro* study has recently shown that a pre-treatment with *Bacillus subtilis* spores before a sodium arsenite-induced oxidative stress allows human keratinocytes to keep normal levels of intracellular ROS, GSH and lipid peroxidation^23^. Spores have been proposed to contribute to cell protection by inducing the expression of antioxidant enzymes through activation of the nuclear translocation of the E2-related factor 2 (Nrf2). In the nucleus, Nrf2 binds to *cis*-acting Antioxidant Response Elements, present in the promoter region of genes coding for antioxidant/detoxifying enzymes, activating transcription initiation^3,23^.

In search for new food additives able to counteract ROS accumulation, we analyzed the potential antioxidant activity of spores of a human isolate of *Bacillus megaterium* (SF185 strain) and used as model systems Caco-2 cells exposed to hydrogen peroxide and Dextran sulfate sodium (DSS)-treated mice.

## Results

### Antioxidant activity of *B*. *megaterium* spores *in vitro*

**Figure 1:**
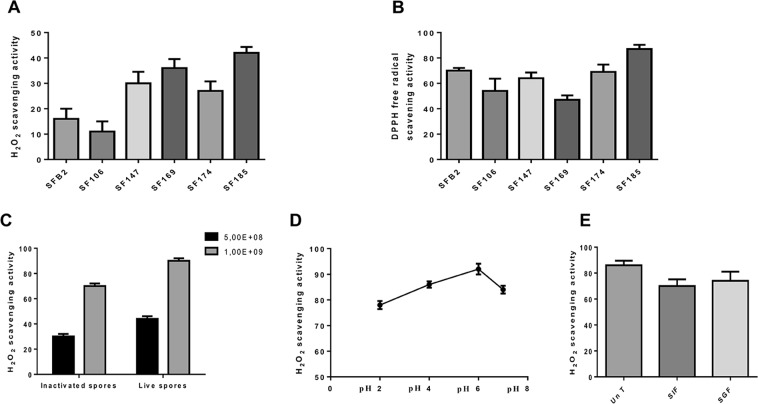
Antioxidant activity of spores produced by human gastrointestinal tract isolates. (**A**) Hydrogen peroxide scavenging activity of 5.0 × 10^8^ spores of *B*. *subtilis* (SFB2 and SF106), *B*. *pumilus* (SF147), *B*. *licheniformis* (SF169), *B*. *clausii* (SF174) and *B*. *megaterium* (SF185) was measured after 30 minutes of incubation and reported as % of H_2_O_2_ removed respect to the control. (**B**) DPPH radical scavenging activity of 1 × 10^9^ spores of strains indicated in (**A**). (**C**) Hydrogen peroxide scavenging activity of 5.0 × 10^8^ (light grey bars) and 1.0 × 10^9^ (dark grey bars) live and inactivated spores of *B*. *megaterium* SF185. (**D**) Effect of low pH conditions and (**E**) of SIF or SGF treatments on the H_2_O_2_ scavenging activity scavenging activity of SF185 spores. Data are mean of three independent experiments ± SE (n = 5).

In order to identify new potential *Bacillus* probiotic strains with antioxidant activity, we compared spores of six previously characterized human isolates of *B*. *subtilis* (SFB2 and SF106), *B*. *pumilus* (SF147), *B*. *licheniformis* (SF169), *B*. *clausii* (SF174) and *B*. *megaterium* (SF185)^24^ for their antioxidant ability. Spores of all six strains were able to scavenge ROS, with spores of strains SF147, SF169 and SF185 performing better than the others in scavenging H_2_O_2_ (Fig. 1A) and spores of strains SFB2, SF147, SF169 and SF185 performing better in scavenging free radicals (Fig. 1B). Vegetative cells of all six strains were also tested and all showed antioxidant activity (Supplementary Fig. S1). For its strong activity against both H_2_O_2_ (Fig. 1A) and free radicals (Fig. 1B), spores of SF185 were selected and used for all further experiments of this study.

The antioxidant activity increased by increasing the number of spores used in the assay and was not affected by heat-inactivation of SF185 spores as autoclaved spores showed a H_2_O_2_-scavenging activity almost similar to that of live spores (Fig. 1C), suggesting that the activity was either due to thermo-stable enzymes or, more likely, to non-enzymatic molecules.

While it is well-established that ingested spores transit through the stomach with no effects on their viability^25^, it is not known whether spore associated antioxidant molecules are affected by the exposure to the low pH conditions typical of the stomach. Therefore, we analysed the stability of the antioxidant activity of the spores at various pH conditions and in the presence of simulated gastric or intestinal fluids (Fig. 1D,E). To this aim, 1.0 × 10^10^ spores of SF185 were incubated for 1 hour either at various acidic conditions or in simulated gastric or intestinal fluid (SGF or SIF, respectively) and the antioxidant activity was assayed as reported in the Materials and Methods section. As shown in Fig. 1D, spores were able to remove hydrogen peroxide at all the pH conditions tested. At pH 2.0 as at pH 7.0, spores were still able to remove almost 80% of H_2_O_2_, while the highest activity was observed at pH 6.0. When incubated in either SGF or SIF, spores showed only a slight decrease of the antioxidant activity (Fig. 1E).

### Antioxidant activity of SF185 spores on *Caco*-*2* cells

Our next step was to verify if *B*. *megaterium* SF185 spores were able to exert antioxidant activity also on cultured cells. Therefore, we used Caco-2 cells, a well characterized intestinal *in vitro* model and measured the intracellular ROS levels by an indirect fluorescent approach (Materials and Methods section). As previously reported for *B*. *subtilis* spores^23^, also SF185 spores did not germinate after 24 hours of incubation in DMEM at 37 °C under CO_2_ pressure (not shown).

**Figure 2:**
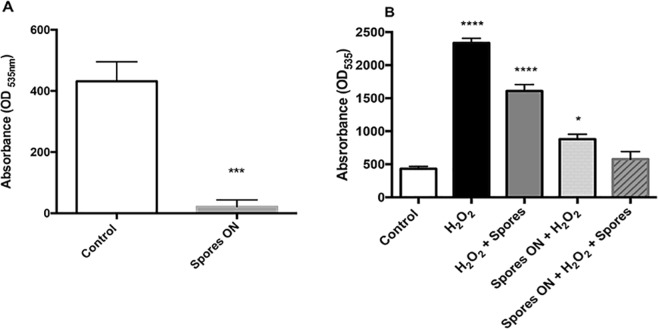
Intracellular ROS analysis. (**A**) Caco-2 cells were grown for 18 hours in presence (grey bar) or not (white bar, control) of spores at 1:1 ratio with cells. Cells were then washed from spores and Intracellular ROS measured by DCFDA assay. (**B**) Caco-2 cells were grown for 18 hours in absence (1,2,3 bars) or presence (4, 5 bars) of spores at 1:1 ratio with cells. Cells were then washed from spores and treated with a DCFDA solution, then washed and challenged (2, 3, 4 and 5 bars) or not (white bar, control) for 2 hours with 100 µM H_2_O_2_ in presence (3, 5 bars) or absence (2, 4 bars) of spores at 1:1 ratio with cells. Data are expressed as means ± SE (n = 3). Analysis of variance has been performed: by unpaired two-tailed t test (**P = 0.0005) in (**A**) and by One-way ANOVA followed by Bonferroni post-test (*P < 0,05 compared with control ****P < 0,0001 compared with control) (**B**).

As Fig. 2A shows, the level of intracellular ROS was drastically reduced after an overnight (ON) incubation (grey bar), with a 95% reduction in ROS accumulation. The protective effect exerted by spores was also observed when cells were subjected to oxidative stress by H_2_O_2_: treatment with 100 μM H_2_O_2_ for 2 hours in the presence of a ratio spores/cells of 1:1 induced a decrease of ROS production (Fig. 2B). Interestingly, the effect was more pronounced when Caco-2 cells were pre-incubated ON with spores, washed to eliminate the spores and then challenged with H_2_O_2_ for 2 hours, indicating that spores can exert a preventive protective effect. Accordingly, if spores were re-added during the H_2_O_2_ treatment following the ON pre-incubation with spores, there was a further slight reduction of ROS production (Fig. 2B).

**Figure 3:**
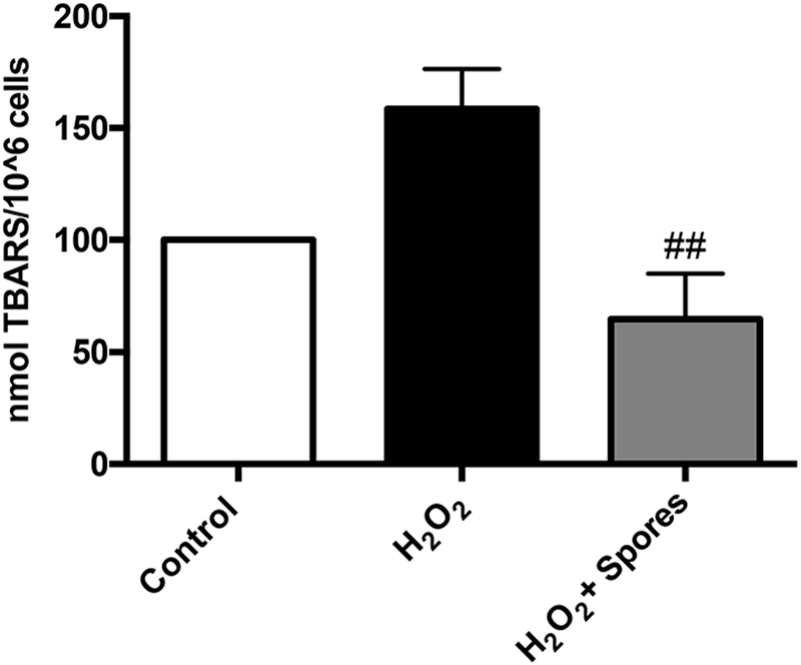
Effects of spores on lipid peroxidation in Caco-2 cells. Caco-2 cells were serum-starved for 2 hours and then treated with 100 µM H_2_O_2_ (black bar) or 100 µM H_2_O_2_ and spores at 1:1 ratio cells/spores (grey bar) for an additional 2 hours. Data are expressed as percentage of untreated cells (white bar, control) set at 100 and presented as mean ± SE (n = 4). Analysis of variance has been performed by One-way ANOVA followed by Bonferroni post-test. ^##^P < 0,01 compared with H_2_O_2_.

Next, we analyzed ROS-induced oxidative damages through evaluation of lipid peroxidation of the Caco-2 cell membranes. To this purpose, cells were incubated for 2 hours with a 1:1 spores/cells ratio, in the presence of 100 µM H_2_O_2_. The level of lipid peroxidation was measured as described in Materials and Methods section. As shown in Fig. 3, the TBARS levels, an indirect measurement of lipid peroxidation, was markedly reduced in samples treated with spores with respect to H_2_O_2_-treated cells, confirming that spores are able to exert antioxidant activity against both exogenous and endogenous ROS.

**Figure 4:**
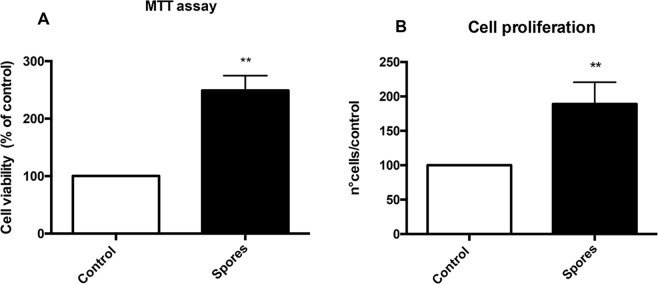
Effects of spores on Caco-2 cells: MTT (**A**) and cell proliferation (**B**) assays. (**A**) Caco-2 cells were incubated for 24 hours with spores at 1:1 ratio. Data are expressed as percentage of control (no spores) (white bar) set at 100 and presented as mean ± SE of three independent experiments. Statistical significance was assessed by unpaired two-tailed t test (**P = 0.0045). (**B**) Caco-2 cells were incubated for 24 hours with spores at 1:1 ratio. Data of cell proliferation are reported as percentage of control (no spores) (white bar) set at 100 and presented as mean +/−  SE of three independent experiments. Statistical significance was assessed by unpaired two-tailed t test (**P = 0.0083).

To further analyze the effects of spores on Caco-2 cells, we asked whether they can affect cell viability. Therefore, an MTT assay was conducted following a 24 hours incubation of Caco-2 cell with *B*. *megaterium* SF185 spores at 1:1 spores/cells ratio. As previously shown for *B*. *subtilis*^23^, also SF185 spores caused an increase of the MTT index values for Caco-2 cells (Fig. 4A). The results of Fig. 4A could be either due to an increase of the cell metabolic activity or to an increase in the cell number. To discriminate between the two possibilities, we performed a cell proliferation analysis with Caco-2 cells incubated for 24 hours with or without spores at 1:1 ratio of spores/cells. A 1.9-fold increase in the cell number was observed in the presence of spores (Fig. 4B), indicating that SF185 spores stimulate Caco-2 cell proliferation, as previously reported for *B*. *subtilis* spores^23^.

### Antioxidant activity of SF185 spores in mouse model

**Figure 5:**
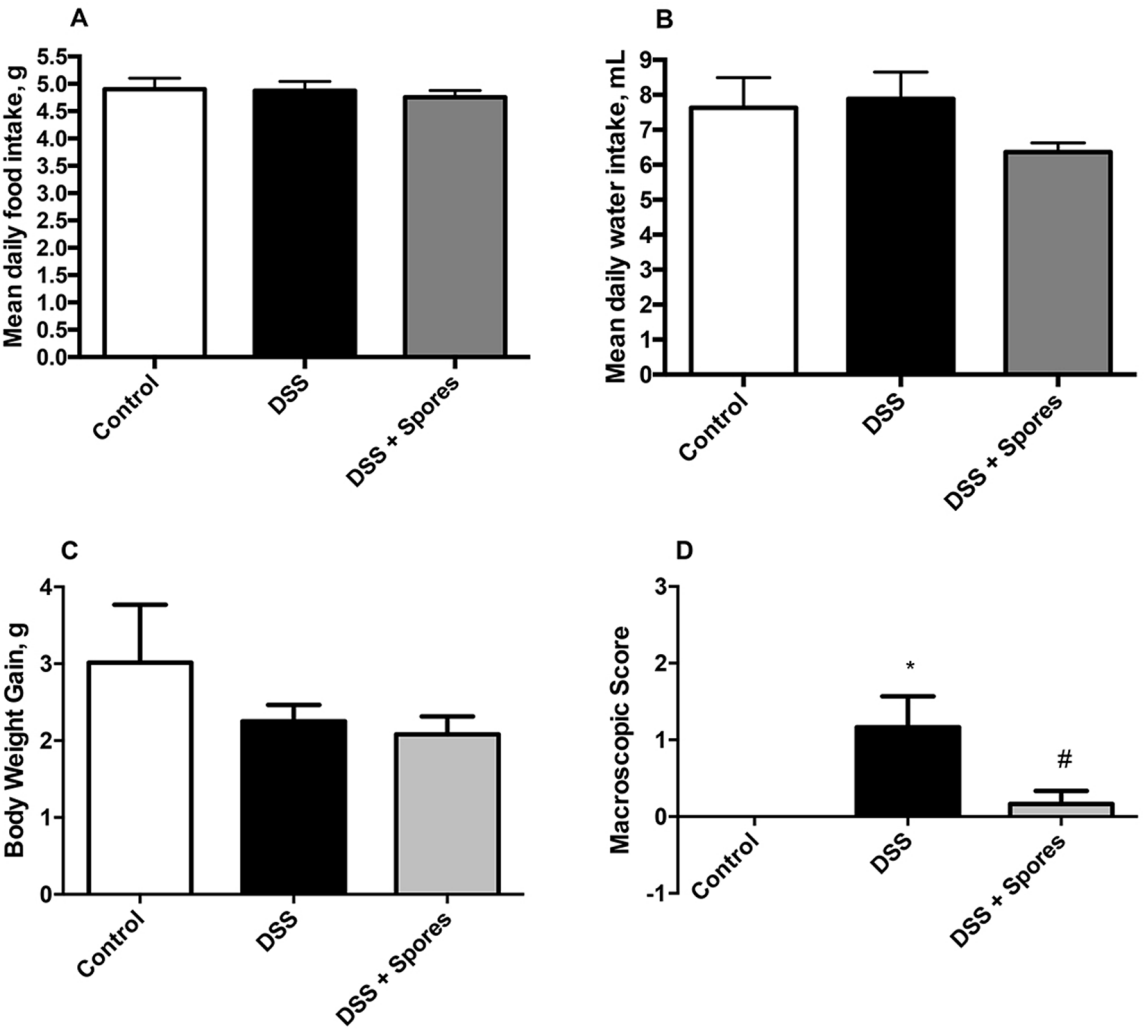
Macroscopic effects of DSS *in vivo*. (**A**,**B**) Food and water intake and (**C**) body weight gain were monitored daily while (**D**) macroscopic damage scores were assessed on the day of euthanasia. In all panels, untreated mice (control, white bars), mice treated with DSS without (black bars) or with spores (grey bars). Values are reported as means with their SE (n = 6). Analysis of variance has been performed by One-way ANOVA followed by Bonferroni post-test. *^,#^P < 0,05 compared with control.

The antioxidant activity of SF185 spores was also tested *in vivo*, by using a mouse model of DSS-induced colitis^26^. DSS addition to drinking water did not influence food, water intake or body weight gain, nor did the administration of spores (Fig. 5A–C). In addition, the induction of colitis by DSS treatment and the protective effect of SF185 spores were analysed determining the macroscopic score (Fig. 5D) that revealed a strong disease activity index in DSS-challenged mice markedly reversed by the spore treatment (Table 1).

**Table 1:** Scoring criteria applied to the macroscopic injuries in the mice colon.

Score	Rectal bleeding	Rectal prolapse	Stool consistency	Blood in the stool samples
0	negative	negative	normal	normal
1	red	signs of prolapse	soft	red
2	dark red	visible prolapse	soft	dark red
3	gross bleeding	extended prolapse	diarrhea	black

**Figure 6:**
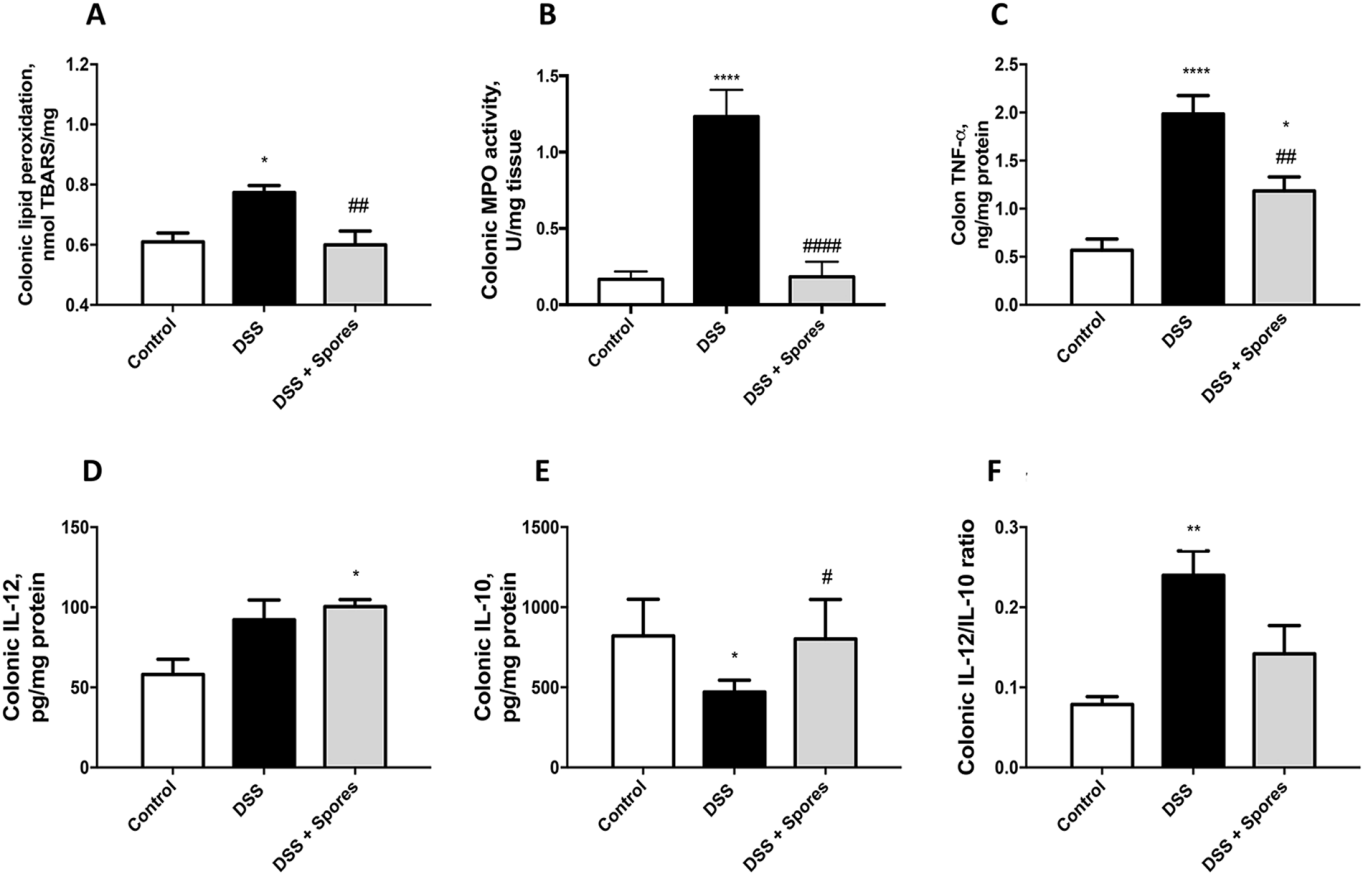
Effects of spores on oxidation, inflammation and cytokines levels. (**A**) Lipid peroxidation, (**B**) MPO activity, (**C**) TNF-alpha levels, (**D**) IL-12 levels and (**E**) IL-10 levels were evaluated on colonic tissue samples collected after euthanasia. (**F**) The IL-12/IL-10 ratio is a marker of disease severity. Values are reported as means with their SE (n = 6). Analysis of variance has been performed by One-way ANOVA followed by Bonferroni post-test. *P < 0,05 compared with control, ****P < 0,0001 compared with control, **P < 0,01 compared with control, ^##^P < 0,05 compared with DSS, ^####^P < 0,0001 compared with the DSS, ^##^P < 0,01 compared with the DSS.

The antioxidant protective effect of SF185 spores was also confirmed in colon samples of DSS-treated mice by evaluating lipid peroxidation, MPO activity and the concentration of the inflammatory cytokine TNFα and the IL12/IL10 cytokines ratio (Fig. 6). As Fig. 6A shows, lipid peroxidation increased in colon samples of mice treated with DSS and was restored to control levels in mice treated with spores. Also, other common inflammation markers, such as colonic TNFα, IL12/IL10 ratio (two cytokines with inflammatory and anti-inflammatory activities, respectively)^27,28^ and MPO activity were restored in spore treated mice (Fig. 6B–F). Overall, these *in vivo* experiments indicate that treatment with SF185 spores is able to protect from a DSS induced colitis with a strong impact on the levels of colon inflammation.

**Figure 7:**
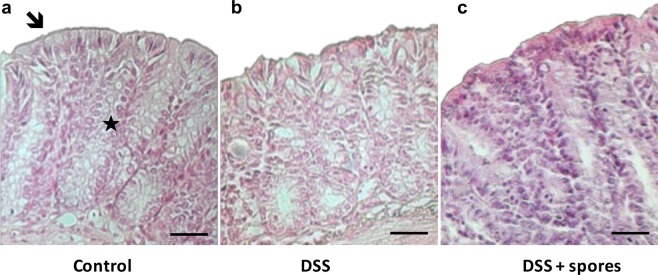
Hematoxylin/eosin staining. Representative photomicrographs of colon histology observed in the three experimental mice groups. (**a**) In control mice the colon tissue showed a normal mucosa with intact crypts (★) and surface epithelium (); (**b**) in colon of DSS-treated the mucosa appeared with epithelial erosion and partial disappearance of crypts; (**c**) ingestion of SF185 spores reduced significantly the severity of inflammation induced by DSS. Scale bar: 40 μm.

To confirm the ability of the SF185 spores to exert a beneficial effect, the morphology of mice intestinal tissues was analyzed by histological analysis (see Materials and Methods section)^26^. In particular, to evaluate the DSS-induced tissue damages, the transversal sections of embedded colon tissues were stained with Hematoxylin&Eosin and then observed at the light microscope (Fig. 7). As expected, the DSS treatment caused severe damage of intestinal tissues as shown by epithelial erosion and alteration of crypts morphology (Fig. 7a,b). Treatment with SF185 spores prevented the damage maintaining the colon integrity and reducing the mucosal inflammation (Fig. 7c).

## Discussion

Several bacterial spore formers, mostly of the *Bacillus* genus, are widely used as probiotics and have been on the market for human or animal use for decades in many countries^21^. The biotherapeutic potential of these bacteria is based on their ability to interact with the internal milieu of the host by the secretion of a variety of effector molecules^29^. While the efficacy as probiotic of the vegetative form of *Bacillus* spp has been analysed in several *in vitro* and *in vivo* animal models, there are only few reports dealing with the health benefits of their spore form. Only recently, the protective effect of spores of a laboratory strain of *B*. *subtilis* against oxidative damages in human epidermal keratinocytes has been reported^23^.

In this context, we searched for potential *Bacillus* probiotic strains with antioxidant activity among human intestinal isolates and identified *B*. *megaterium* SF185 as the most promising strain. Our results indicate that spores of this strain have a high intrinsic and protective antioxidant activity *in vitro* and *in vivo* on a mice model. While vegetative cells of SF185 have been previously shown to produce and secrete bioactive molecules that exert cytoprotective functions on colon epithelial cells^30^, spores of this strain have never been tested before.

It is well known that the spore surface contains enzymes of the Kat and SOD families^31,32^; however, we show that autoclaved spores have the same activity of live spores. Therefore, either thermostable enzymes are present on the surface of SF185 or non-enzymatic antioxidant molecules are involved.

The spore antioxidant activity is stable at low pH or in simulated gastric and intestinal conditions suggesting that SF185 spores could maintain the ability to remove H_2_O_2_ and act as antioxidants also upon the ingestion and transit through the gut (Fig. 1D,E).

Moreover, the effects observed on Caco-2 cells clearly suggests that spores can act as ROS scavengers reducing free oxidants. Interestingly, this effect was also exerted against oxidants produced by the physiological metabolic activity of eukaryotic cells as observed when spores were incubated with cells not subjected to any oxidative insult (Fig. 2A). This is further supported by the increased metabolic activity and proliferation rate of spore-supplemented Caco-2 cells not treated with H_2_O_2_ (Fig. 4). A clear protective effect was also observed when spores were incubated with Caco-2 cells for 18 hours and then removed before the treatment of cells with H_2_O_2_; this result indicates that spores pre-incubation induces a reduction of oxidants or catabolites produced during cell proliferation, ameliorating growth conditions and allowing cells to better resist to ROS induced damages (Fig. 2B).

In support of our observation it has been recently reported that spores of a different Bacillus species, *B*. *subtilis*, induce the nuclear translocation of the Nrf-2 factor with the subsequent activation of stress response genes^23^. Interestingly, Nrf2 transcription factor was shown to play pivotal roles in the regulation of cellular redox homeostasis and cell proliferation^33^. This observation is in agreement with the increase in cell proliferation that we observed in our experimental conditions. We speculated that a similar mechanism could be induced by *B*. *megaterium* spores in Caco-2 cells. Addressing this point will be a challenging task of a future work.

Experiments in mice in which colitis was induced by treatment with DSS show the protective effects of pre-dosing the animals with SF185 spores. In particular, the spore treatment protects against weight loss and colon damage through reduction of inflammation and of oxidative stress (Figs 5–6). In addition, the intestinal tissue integrity was preserved by spores treatment as shown by the reduction of typical histological changes such as epithelial shallow erosions and crypt degenerations that occur in DSS-induced colitis (Fig. 7).

While additional experiments are needed to clarify the precise mechanism of action, our data strongly suggest that spores of *B*. *megaterium* SF185 represent a new potential promising probiotic and that administration of spore-based products may have preventive efficacy in oxidative stress induced damages.

## Methods

### Bacterial strains

Sporulation of *Bacillus* strains was induced by the exhaustion method^34^. After 30 hours of growth in Difco Sporulation medium (DSM) at 37 °C with vigorous shaking, spores were collected, washed three times with distilled water and purified by gastrografin gradient as described before^34^. Spore counts were determined by serial dilution and plate-counting.

### Hydrogen peroxide scavenging assay

The hydrogen peroxide stability was meausered by following absorbance at 240 nm of 1 ml of fresh hydrogen peroxide solution [50 mM Potassium Phosphate Buffer, pH 7.0; 0.036% (w/w) H_2_O_2_] at our experimental condition [temperature 20 °C, pH 7.0]. Since the H_2_O_2_ solution readings were more stable after 30 minutes (Supplementary Fig. S2), we used this interval time to further experiments. Quantitative determination of H_2_O_2_ scavenging activity of spores was measured by the loss of absorbance at 240 nm as previously described by Beers and Sizer^23,35^.

Briefly, 5 × 10^8^ or 1 × 10^9^ spores of selected strains were incubated at 20 °C in 1 ml of hydrogen peroxide solution [50 mM Potassium Phosphate Buffer, pH 7.0; 0.036% (w/w) H_2_O_2_]. After 30 minutes, aliquotes were centrifuged for 1 min at 13000 g to remove the spores and the hydrogen peroxide concentration in the supernatant was determined by measuring the absorbance at 240 nm. The percentage of peroxide removed was calculated as follows:

$${{\rm{H}}}_{{\rm{2}}}{{\rm{O}}}_{{\rm{2}}}\,{\rm{removed}}\,( \% )=(1-\frac{{A}_{samples}}{{A}_{control}})\times 100$$

where *A*_*contro*l_ is the absorbance of 1 ml of hydrogen peroxide solution.

### DPPH Assays

The α,α-diphenyl-β-picrylhydrazyl (DPPH) free radical scavenging method was used to evaluate the potential antioxidant activity of spores^36^. 1×10^9^ spores of selected strains were incubated in a final volume of 1 ml of 100% methanol containing 0.1 mM of freshly prepared DPPH (giving absorbance ≤ 1.0). The reaction was allowed to proceed for a maximum time of 30 minutes at 25 °C, in any case till completion, and followed at λ C,515 nm. The DPPH free radical scavenging activity was calculated according to the following equation:

$${\bf{DPPH}}\,{\rm{radical}}\,{\rm{scavenging}}\,{\rm{activity}}\,( \% )=(1-\frac{{A}_{samples}}{{A}_{control}})\times 100$$

where *A*_*sample*_ is the absorbance of the reacted mixture of DPPH with the extract sample, and *A*_*control*_ is the absorbance of the DPPH solution.

### pH-stability assay

Spores were incubated at 37 °C for 1 hour in following buffers: 0.1 M Glycine-HCl pH 2.0; 0.1 M citrate-phosphate pH 4.0 and pH 6.0, or 0.1 M HEPES pH 7.0. After incubation, the samples were centrifuged for 5 min. at 13000 × g. The hydrogen peroxide scavenging activity of the samples was measured following the protocol described above.

### **I**ntestinal-stability assay

Spores were incubated for 1 hour at 37 °C in 100 μl of simulated gastric juice (SGF) [1 mg of pepsin (porcine stomach mucosa; Sigma) for ml of 10 mM HCl; pH 2.0] or small intestine fluid (SIF) [1 mg of pancreatin (porcine pancreas; Sigma) for ml and 0.2% bile salts (50% sodium cholate-50% sodium deoxycholate; Sigma); pH 6.8]. To remove the proteases contained in SIF and SGF, after incubation, spores were washed, collected by centrifugation (10 min at 13000 × g) and then assayed for the hydrogen peroxide scavenging activity.

### **C**ell culture conditions, MTT assay and cell proliferation analysis

Human colon adenocarcinoma Caco-2 cells (ATCC HTB-37) were routinely cultured at 37 °C under 50% confluence in a humidified 5% CO_2_ incubator in Dulbecco’s Modified Eagle Medium (DMEM, Gibco) Supplemented with 10% (v/v) FBS (Gibco), 1% penicillin-streptomycin (Gibco), 1% L-glutamine (Gibco)^37,38^. The cellular energy capacity of the Caco-2 cells was assessed using the MTT assay (Sigma-Aldrich). It is based on the reduction of the tetrazolium ring of 3-(4,5-dimethylthiazol-2-yl)-2,5-diphenyltetrazolium bromide (MTT) by mitochondrial dehydrogenases, yielding a purple dye (formazan), which can be measured spectrophotometrically; the amount of formazan produced is proportional to the number of metabolically active cells. Caco-2 cells were seeded in 12-well plates (0.9 × 10^5^ cells/well). Cells were then treated with 1:1 ratio of cells/spores. Cells were incubated for 3 h at 37 °C with a 1X MTT solution diluted in DMEM without Phenol Red, supernatant was removed, and acidic isopropanol 0.01 N was added to each well to dissolve insoluble formazan crystals formed. The absorbance of the samples was measured at a 570 nm using a microplate reader (Multiskan spectrum, Thermo)^39,40^.

For cell proliferation analysis, Caco-2 cells were seeded in 12-well plates at a density of 0.9 × 10^5^ cells/well and incubated for 24 hours in the presence of spores 1:1 ratio of cells/spores. After 24 h incubation cells were collected and the number of cells in each experimental point was counted with the Scepter-Millipore counter (Handheld Automated Cell Counter).

### Intracellular ROS evaluation

Intracellular ROS levels were quantified by a DCFDA assay (Abcam), based on the cell permeant reagent 2’,7’- dichlorofluorescin diacetate (DCFDA), a fluorogenic dye that measures reactive oxygen species activity within the cell. After diffusion into the cell, DCFDA is deacetylated by cellular esterase to a non-fluorescent compound, which is later oxidized by ROS into 2’, 7’-dichlorofluorescein (DCF). DCF is a highly fluorescent compound which can be detected by fluorescence spectroscopy, and the fluorescence intensity is proportional to intracellular ROS produced. Caco-2 cells were seeded in 96-well white walled plates (Invitrogen, Thermo Fisher Scientific) at a density of 2.5 × 10^4^ cells/well. Cells were incubated overnight with or without (control) 2.5 × 10^4^ spores/well and then extensively washed with a saline buffer (Buffer 1×). Then a solution of DCFDA (25 µM) was added to each well and cells were incubated for 45 min in dark at 37 °C. When indicated, cells were subjected to chemical-induced oxidative stress with H_2_O_2_ for 2 hours with or without spores at 1:1 ratio spores/cells. Afterwards, fluorescence intensity was measured at λ excitation and λ emission of 485 nm and 535 nm, respectively, in a plate reader (Synergy 4, BioTek). The number of residual spores after rand emoval washing steps was calculated by plating on LB agar plates and it resulted to be less than 10%.

### Lipid peroxidation in Caco-2 cells

Lipid peroxidation products [thiobarbituric acid reactive substances (TBARS) also known as malondialdehyde-equivalents (MDA-equivalents)] from Caco-2 cells were measured by the thiobarbituric acid colorimetric assay, according to Fernandes *et al*.^13,40^. Briefly, Caco-2 cells were seeded in 6-well plates at density of 2.5 × 10^5^ cells/well. Cells were serum-starved for 2 h and then co-treated with H_2_O_2_ 100 µM and spores at 1:1 ratio of cells/spores in complete medium for an additional 2 h. Then, the medium was removed, cells were washed with PBS, counted and centrifuged at 500 g for 5 min at 25 °C. After removal of supernatant, 0.5 ml of ice-cold 40% trichloroacetic acid and 0.5 ml of 0.67% of aqueous thiobarbituric acid were added to the pellet. The mixtures were heated at 90 °C for 15 min, then cooled in ice for 10 min, and centrifuged at 800x g for 10 min. The supernatant fractions were collected, and lipid peroxidation estimated spectrophotometrically at 530 nm. The amount of TBARS formed was calculated using a molar extinction coefficient of 1.56 × 10^5^/mol/cm and expressed as nmol TBARS/10^6^ cells.

### Mouse model and experimental design

Eight-week-old C57BL/6 mice were housed in standard animal cages under specific pathogen-free conditions. The mice were maintained in an environment of constant temperature and humidity with a 12-hours light-dark cycle and were given free access to food and water. Treatment, housing, and euthanasia of the animals met the guidelines set by the Italian Health Ministry. *Comitato Etico*-*Scientifico per la Sperimentazione Animale* of the University “Federico II” of Naples approved this study (Prot. 2013/0068806). All experiments were performed in accordance with relevant guidelines and regulations.

Experimental colitis was induced in the C57BL/6 mice by added DSS (2%, wt:vol) in drinking water ad libitum from day 2 to day 6, followed by DSS-free water for 2 days. Mice were randomly allocated into the three following groups (n  m6 per group): 1) untreated mice (Control group); 2) mice receiving DSS in water (DSS group) for 5 days; 3) mice receiving DSS in water for 5 days and 1 × 10^9^ spore by gavage, starting from two days before the DSS treatment, until two days after DSS treatment (DSS spores group). During the treatments, body weight, food and water intake were monitored daily.

### Evaluation of experimental colitis

After mice euthanasia, the macroscopic score was evaluated according to the scheme of Table 1.

The macroscopic score was determined by combining the scores from these 4 categories and dividing the resulting number by 4.

### Lipid peroxidation in colon samples

Homogenate from descending colon were assayed for lipid peroxidation as described above. The amount of TBARS formed was calculated using a molar extinction coefficient of 1.56 × 10^5^/mol/cm and expressed as nmol TBARS/mg tissue

### Determination of TNF-α, IL-12 and IL-10 in ascending colon samples

TNF-α and IL-12/IL-10 concentrations in protein extracts from ascending colon descending were determined using an enzyme linked immunosorbent assay (R&D Systems, MN, USA and ThermoFisher Scientific, lL USA, respectively) according to manufacturer instructions. Briefly, for TNF-**α** the wells of a microtitre plate were coated with 100 µl of mouse anti-rat TNF-**α** (4 µg/ml) in PBS (137 mM NaCl; 2.7 mM KCl; 8.1 mM Na_2_HPO_4_;1.5 mM KH_2_PO_4_, pH 7.4), and incubated overnight at 25 °C. The antibody excess was then removed by washing with Wash Buffer (containing 0.05% (v/v) Tween 20 in PBS, pH 7.4), and the remaining sites on the plate were blocked with reagent diluent (PBS containing 1% BSA) (1 h, room temperature). After extensive washing, 100 μl of samples (1:2–1:10 dilution in reagent diluent) were added to the wells and incubated for 2 hours at room temperature. After further washing, the wells were incubated with biotinylated goat anti-rat TNF-**α** (225 ng/ml in reagent diluent) followed by treatment with Streptavidin-HRP (1:200 dilution; 1 h, room temperature). For IL-12/IL-10 determination, 50 μl of samples (1:2 dilution in reagent diluent for IL-12, undiluted for IL-10) were added in the wells of a microtiter plate and incubated for 1 h at 20–25 °C for IL-12 and for 3 h at 20–25° for IL-10. After 3 washing with Wash Buffer (provided by the kit), the wells were incubated with 100 μL for IL-12 and 50 μL for IL-10 of Biotinylated Antibody Reagent (BAR) for 1 hours at 20–25 °C. After 3 washing with wash buffer, the wells were treated with Streptavidin-HRP (1:400 dilution; 30 minutes, 20–25 °C). For both TNF-α and IL-12/IL-10 determination, peroxidase-catalyzed color development from tetramethylbenzidine was measured at 450 nm.

### Myeloperoxidase (MPO) activity in descending colon

MPO activity was assessed in colon samples as previously described^40^. Briefly, tissue samples (100 mg) were homogenized in 1 ml of hexadecyltrimethylammonium bromide (HTAB) buffer (0.5% HTAB in 50 mM phosphate buffer, pH 6.0) and centrifuged at 13400 × g for 6 minutes at 4 °C. MPO activity was measured spectrophotometrically: 10 µl of supernatant were combined with 200 µl of 50 mM phosphate buffer, pH 6.0, containing 0.167 mg/ml 0-dianisidine hydrochloride and 1.25% hydrogen peroxide. The change in absorbance at 450 nm was measured and one unit of MPO activity was defined as that degrading 1 µmol of peroxide per minute at 25 °C^41^.

### Histological analysis

For histological analysis, distal colonic samples (n: 3 for each experimental group) were embedded in paraffin after fixation in Bouin solution for 48 h and cut as transversal sections of 6 µm. To study the tissue structure, the transversal sections were stained with Hematoxylin and Eosin (H&E) and analysed at the light microscope using the 20x objective lens. Images were acquired by a Kontron Electronic Imaging System KS300 (Zeiss, Germany).

### Statistical analysis

All data are expressed as means of independent experiments ± standard errors (SE). The analysis of variance was carried out by using One-way ANOVA or by two-tail unpaired t-test. The statistical analysis of *in vitro* and *in vivo* experiments was performed with the use of Graph-Pad Prism (Graph-Pad Software).

### Ethical standards statement

All experimental procedures involving animals were approved by “Comitato Etico-Scientifico per la Sperimentazione Animale” of the University “Federico II” of Naples.

## Supplementary information

Supplementary figures
