# Supplementary material for: Bacillus megaterium SF185 spores exert protective effects against oxidative stress in vivo and in vitro
Source: Sci Rep. 2019 Aug 19;9:12082. doi: 10.1038/s41598-019-48531-4 (PMC6700169; doi:10.1038/s41598-019-48531-4)
Supplement: Supplementary file 1 — Supplementary figures [file 41598_2019_48531_MOESM1_ESM.pdf]

Bacillus megaterium SF185 spores exert protective effects against oxidative stress in vivo and in vitro

Arianna Mazzoli °, Giuliana Donadio°, Mariamichela Lanzilli, Anella Saggese, Andrea Maria Guarino, Miriam Rivetti, Raffaella Crescenzo, Ezio Ricca, Ida Ferrandino, Susanna Iossa, Alessandra Pollice* and Rachele Isticato*

Department of Biology, Federico II University, Naples, Italy
* corresponding authors:
apollice@unina.it
isticato@unina.it

° These authors contributed equally to the work.

SUPPLEMENTARY FIGURES

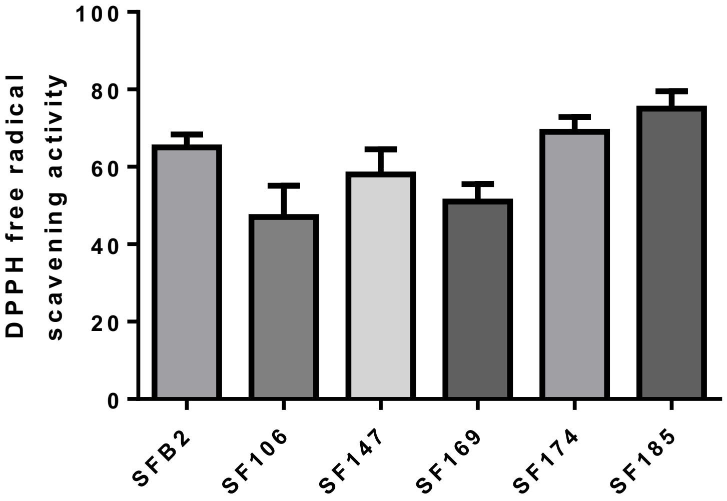

Figure S1. Free radical scavenging activity of vegetative cells of intestinal isolates. The gut *Bacillus* strains were inoculated into Luria Bertani broth and incubated at 37°C for 18 h. $1x10^9$ cells of each strain were tested for the ability to reduce free radicals by DPPH assay (see Materials and Methods section). Results are expressed as the mean ± standard error of means (n = 3).

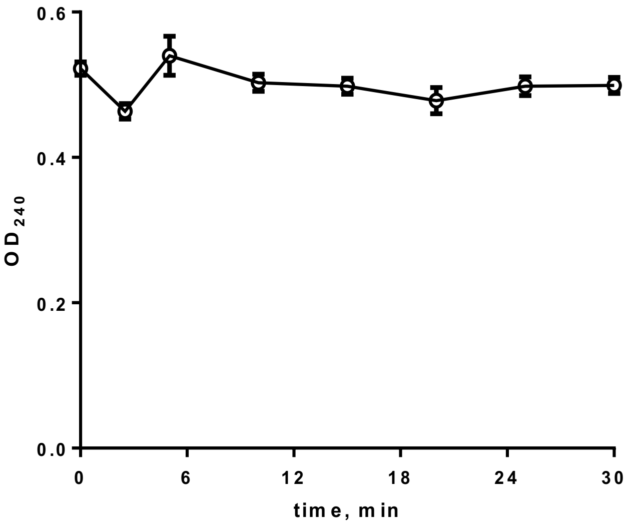

Figure S2. Stability of hydrogen peroxide in the experimental conditions.
The OD_{240nm} corresponding to H_2O_2 decay was measured at 5 min intervals for 30 min (see Materials and Methods section). Each reading represents the mean of three technical replicates and the vertical lines represent the standard errors of the mean.
